# Oxidative Damage Markers Are Significantly Associated with the Carotid Artery Intima-Media Thickness after Controlling for Conventional Risk Factors of Atherosclerosis in Men

**DOI:** 10.1371/journal.pone.0119731

**Published:** 2015-03-25

**Authors:** Jin-Ha Yoon, Jang-Young Kim, Jong-Ku Park, Sang-Baek Ko

**Affiliations:** 1 Department of Preventive Medicine, Wonju College of Medicine, Yonsei University, Wonju, Korea; 2 Department of Cardiology, Wonju College of Medicine, Yonsei University, Wonju, Korea; 3 The Institute for Occupational Health, Yonsei University College of Medicine, Seoul, Korea; 4 Institute of Genomic Cohort, Yonsei University, Wonju, Korea; University of Catanzaro Magna Graecia, ITALY

## Abstract

**Background:**

This study aimed to assess the association between oxidative damage markers and carotid artery intima-media thickness (CIMT) after controlling for conventional risk factors of atherosclerosis in multiple logistic regression models.

**Methods and Findings:**

Fifty-one case male participants (CIMT ≥ 0.9 mm) were enrolled during their visits to Korean Genomic Rural Cohort Study of Wonju centers between May 1 and August 31, 2011, along with 51 control participants (CIMT < 0.9 mm) selected using frequency matching by age group. The levels of oxidative damage markers, 8-hydroxy-2′-deoxyquuanosine (8-OHdG), malondialdehyde (MDA), and 8-iso-prostaglandin F2α (Isoprostane), were measured. Conditional logistic regression models were used to evaluate relative relationships between the oxidative damage markers and the risk of high CIMT.

**Results:**

The markers of oxidative lipid (Isoprostane and MDA) and DNA (8-OHdG) damage were associated with CIMT after controlling for the conventional risk factors, including age, low density lipoprotein, body mass index, smoking history, alcohol consumption, and metabolic syndrome (ORs [95% CI] for Isoprostane: 3^rd^ tertile, 8.47 [2.59-27.67]; for MDA: 3^rd^ tertile, 8.47 [2.59-27.67]; for 8-OHdG: 3^rd^ tertile, 5.58 [1.79-17.33]). When all the oxidative damage markers were incorporated in the same logistic regression model, only Isoprostanewas significantly related to CIMT (OR [95% CI]: 4.22 [1.31-13.53] in 2^nd^ tertile and 14.21 [3.34-60.56] in 3^rd^ tertile).

**Conclusions:**

In this nested case-control study, the oxidative damage markers of lipid and DNA were associated with CIMT even after controlling for the conventional risk factors of cardiovascular diseases.

## Introduction

Atherosclerosis and endothelial dysfunction are considered underlying mechanisms of cardiovascular disease [1]. Numerous studies examining the complex pathophysiological mechanisms associated with cardiovascular disease have revealed a critical role of oxidative stress in the development of atherosclerosis [2].

Oxidative stress is caused by overproduction of reactive oxygen species (ROS) and free radicals beyond the physiological detoxifying capacity of the cells or their ability to repair the resulting damage [3]. Although ROS and free radicals are essential elements of biological systems, such as cell signaling, controlling vascular tone, and generation and degeneration of target cells [4], their high chemical reactivity causes oxidative damage of lipids, DNA, and proteins. However, the direct measurement of free radicals and ROS using electron resonance or spin trapping is very technically challenging and expensive in humans [5]. Therefore, simpler methods that examine the end products of oxidative damage are used to evaluate oxidative stress. For example, 8-hydroxy-2′-deoxyguanosine (8-OHdG) is a marker of damaged DNA [6], whereas malondialdehyde (MDA) and 8-iso-prostaglandin F2α (Isoprostane) are markers of lipid peroxidation damage [4,7]. These forms of oxidative damage are considered key pathologic mechanisms underlying cardiovascular disease [8].

Although there is pathophysiologic evidence indicating that oxidative damage markers are linked to the risk of atherosclerosis [8], human studies utilizing multivariate analysis to control for conventional atherosclerosis risk factors, such as obesity, blood pressure, insulin resistance, lipid profile, smoking history, and alcohol consumption, are relatively rare and controversial [9]. In particular, it has been suggested that there is no independent association between oxidative stress and coronary artery disease when the conventional cardiovascular risk factors are accounted for in statistical models [10]. To resolve this controversy, in the present study we conducted multivariate analyses to investigate the relationship between the oxidative damage markers and the carotid artery intima-media thickness (CIMT) after controlling for the conventional risk factors of atherosclerosis. The results provide new insights into the roles of individual DNA and lipid oxidative damage markers in atherosclerosis.

## Materials and Methods

### Ethics Statement

The current study was nested within the Korean Genomic Rural Cohort Study (KGRC). All participants provided written informed consent for their participation. This study was approved by the Institutional Review Board of Wonju Christian Hospital.

### Study subjects

Male participants were enrolled during their visits to Korean Genomic Rural Cohort Study of Wonju centers between May 1 and August 31, 2011. A medical history questionnaire was used to screen for the exclusion criteria among a total of 214 individuals who visited the cohort centers. We excluded 40 individuals who had a cardiovascular event, e.g., angina, myocardial infarction, and stroke, or a medical history of chronic hepatitis, osteoporosis, kidney disease, asthma, or any malignant disease. Using a CIMT cut-off level of 0.9 mm [11,12], 51 case participants (CIMT ≥ 0.9 mm) and 51 control participants (CIMT < 0.9 mm) were sampled at random using frequency matching by age group (41–50, 51–60, 61–70, and 71–80 years).

### Measurement of anthropometrics, metabolic characteristics, and oxidative damage markers

Comprehensive questionnaires were administered and physical examinations were performed according to standard procedures [13]. A history of regular alcohol consumption was recorded. The subjects were categorized according to their smoking status (current smoker, ex-smoker, and never smoked), and that status was categorized into never vs. ever smoker. The following parameters were obtained from a self-reported questionnaire: medical history of cardiovascular events, e.g., angina, myocardial infarction, and stroke; medical history of chronic hepatitis, osteoporosis, kidney disease, asthma, or any malignant disease; and pharmacological treatment of hypertension (HTN), diabetes mellitus (DM), and dyslipidemia.

Body weight, height, and waist circumference were measured while wearing indoor clothing without shoes. Systolic blood pressure (SBP) and diastolic blood pressure (DBP) were measured twice from the right arm using a standard mercury sphygmomanometer (Baumanometer; USA). Mean SBP and DBP levels were used for data analysis. Venous blood samples were drawn in the morning after overnight fasting and stored at -80°C. Fasting blood glucose (FBG) and insulin (FBI) levels were determined by a glucose oxidase-based assay and double-antibody radioimmunoassay (RIA). The serum concentrations of low-density lipoprotein cholesterol (LDL), high-density lipoprotein cholesterol (HDL), and triglycerides were determined by enzymatic methods (ADVIA 1650; Bayer, USA). All urine samples were collected in ultraviolet-safe urine tube, and were stored at -80°C. The urine samples were clean-up by solid phase extraction with methanol, phosphate butter before analysis. The concentrations of 8-OHdG, Isoprostane, and MDA were measured in spot urine samples using a high-performance-liquid chromatography-triple tandem mass spectrometer (HPLC-MS/MS, Agilent 6410; Agilent). The urinary levels of all oxidative stress damage markers were corrected according to urine creatinine values. The level of urine creatinine was measured by Jaff reaction. The method detection limits and relative standard deviations for accuracy and repeatability, respectively, were 0.053 μg/L and 1.29%/0.90% for 8-OHdG, 0.162 pg/mL and 1.29%/2.10% for Isoprostane, and 0.044 μmol/L and 5.36%/2.10% for MDA. Glomerular filtration rate was calculated by using modification of diet in renal diseases formula [14].

### Ultrasound imaging analysis

Bilateral carotid intima-media thickness (CIMT) on both sides was measured on longitudinal 2-dimentional ultrasonography images recorded with a B-mode ultrasound system (Vivid 7; General Electric Vingmed) with a 12-MHz transducer. Still images of the region near the carotid bifurcation were digitally acquired, and the far walls of the carotid artery were displayed as 2 bright lines separated by a hypoechoic space. CIMT between the leading edge of the first bright line (lumen-intima interface) and the leading edge of the second bright line (media-adventitia interface) was obtained using semi-automated edge-detection software. The values of CIMT exceeding 2 cm were measured within 1 cm from the carotid bulb. The mean maximum value of CIMT on both sides was chosen as the indicator of subclinical atherosclerosis [15].

### Statistical Analysis

Data were expressed as frequencies (%), mean values with standard deviations, and median values with low and high quartiles. The distribution of continuous variables was examined for skewness and kurtosis and triglyceride was logarithmically transformed. We used the *t*-test, Mann-Whitney U test, or chi-square test to compare the differences between the case and control groups. Multiple conditional logistic regression models were tested for the ORs of oxidative damage markers with an adjustment for conventional risk factors, e.g., LDL, body mass index (BMI), smoking history, alcohol consumption, SBP, FBG, chronic kidney diseases and pharmacological treatment of HTN, DM and dyslipidemia. The tertile (T) increment of Isoprostane, 8-OHdG, and MDA was used as an independent variable (Isoprostane [ng/mg creatinine]: 1^st^ T, <0.21; 2^nd^ T, 0.21–0.51; 3^rd^ T, ≥0.51; 8-OHdG [μg/g creatinine]: 1^st^ T, <0.66; 2^nd^ T, 0.66–1.25; 3^rd^ T, ≥1.26; MDA [μmol/g creatinine]: 1^st^ T, <0.10; 2^nd^ T, 0.10–0.20; 3^rd^ T, ≥0.20). Statistical significance was determined at *P* < 0.05 for all comparisons in the current study.

## Results

### Anthropometric and metabolic characteristics of the case and control groups

We used frequency matching according to the age distribution in the case group (Table 1). The smoking history and pharmacological treatment of HTN, DM, and dyslipidemia were similar for the 51 case participants and 51 controls. Various metabolic biomarkers, such as triglycerides, FBG, FBI, SBP, and DBP were also similar between the case and control groups. The serum level of LDL was higher in the case group than in the control group. The levels of all the oxidative damage markers were significantly higher in the case group than in the control group (median values of case vs. control groups, respectively: Isoprostane, 0.50 vs. 0.21, *P* < 0.0001; 8-OHdG, 1.26 vs. 0.74, *P* = 0.0021; MDA, 0.20 vs. 0.11, *P* = 0.0005).

**Table 1:** Demographic and metabolic characteristics of the case and control groups.

	Cases (n = 51)CIMT ≥0.9 mm		Controls (n = 51)CIMT < 0.9 mm		*P* value
***Demographic characteristics***					
Age, n (%)					1.000
40~50	2	(3.92)	2	(3.92)	
51~60	14	(27.45)	14	(27.45)	
61~70	29	(56.86)	29	(56.86)	
71~80	6	(11.76)	6	(11.76)	
Pharmacologic treatments, n (%)					
Hypertension	18	(35.29)	20	(39.22)	0.6821
Diabetes	6	(11.76)	5	(9.08)	0.7496
Dyslipidemia	6	(11.76)	9	(17.65)	0.4016
% of never smoker, n (%)	19	(37.25)	24	(47.06)	0.4239
% of alcohol drinker, n (%)	34	(66.67)	35	(68.63)	0.8324
***Metabolic characteristics***					
Isoprostane / creatinine ratio	0.50	(0.27–0.80)	0.21	(0.15–0.47)	<.0001
8-OHdG / creatinine ratio	1.26	(0.69–1.83)	0.74	(0.45–1.14)	0.0021
MDA / creatinine ratio	0.20	(0.13–0.28)	0.11	(0.08–0.16)	0.0005
Triglyceride (mg/dL)	133.00	(97–177)	128.00	(84–159)	0.2955
Body mass index (kg/m2)	23.98	±2.73	24.20	±2.93	0.6935
Waist circumference (cm)	85.25	±5.78	86.11	±7.71	0.5289
Fasting plasma glucose (mg/dL)	104.92	±24.73	98.75	±14.23	0.1290
Systolic blood pressure (mmHg)	127.48	±14.55	126.88	±14.92	0.8390
Diastolic blood pressure (mmHg)	76.94	±11.72	80.59	±9.55	0.0893
LDL (mg/dL)	117.58	±25.94	106.02	±31.07	0.0453
HDL (mg/dL)	50.02	±9.89	51.65	±12.44	0.4692
MetS proportion (n, %)	17	(33.30)	18	(35.29)	0.8348
eGFR (mL/min/1.73 m^2^)	79.34	x12.55	76.97	±11.28	0.3163

Abbreviations: CIMT, carotid intima media thickness; BMI, body mass index; Isoprostane, 8-iso-prostaglandin F2α; 8-OHdG, 8-hydroxy-2'-deoxyguanosine; MDA, malondialdehyde; MetS, metabolic syndrome; LDL, low density lipoprotein; HDL, high density lipoprotein; eGFR, estimated glomerular filtration rate.

### The relationship between the oxidative damage markers and CIMT after controlling for the conventional risk factors of atherosclerosis in multiple logistic regression models (Table 2)

**Table 2:** Relationship between oxidative damage marker and carotid artery intima media thickness (mm).

			Model I	Model II
			OR (95% CI)	OR (95% CI)
**Isoprostane,ng/mg creatinine**	OR (95% CI)	1st tertile (<0.21)	1.00 -	1.00 -
		2nd tertile (<0.51)	3.80 (1.36–10.59)	4.221 (1.31–13.53)
		3rd tertile (≥0.51)	7.30 (2.50–21.29)	14.21 (3.34–60.56)
	P for trend		<0.001	<0.001
**MDA,μmol/g creatinine**	OR (95% CI)	1st tertile (<0.10)	1.00 -	1.00 -
		2nd tertile (<0.20)	3.08 (1.13–8.42)	4.22 (1.27–13.99)
		3rd tertile (≥0.20)	6.39 (2.24–18.25)	6.46 (1.91–21.83)
	P for trend		0.001	0.002
**8-OHdG,μg/g creatinine**	OR (95% CI)	1st tertile (<0.66)	1.00 -	1.00 -
		2nd tertile (<1.26)	1.34 (0.48–3.78)	0.77 (0.22–2.64)
		3rd tertile (≥1.26)	5.43 (1.86–15.84)	4.45(1.27–15.56)
	P for trend		0.002	0.020

ORs were calculated at level of carotid intima media thickness > 10 mm.

P for trend were calculated by conditional logistic regression models.

Abbreviations: Isoprostane, 8-iso-prostaglandin F2α; 8-OHdG, 8-hydroxy-2'-deoxyguanosine; MDA, malondialdehyde; OR (95% CI), odds ratio (95% confidence interval), OR (95% CI): odds ratio (95% confidence interval).

Model I: not adjusted

Model II: adjusted for age, smoking history, regular alcohol consumptions, systolic blood pressure, fasting blood glucose, low density lipoprotein, glomerular filtration rate and pharmacological treatment of hypertension, diabetes and dyslipidemia.

Compared with the 1^st^ tertile of Isoprostane and MDA values, the 2^nd^ and 3^rd^ tertiles of these oxidative damage markers were associated with an increased risk of high CIMT in model I (ORs [95% CI] for Isoprostane: 2^nd^ tertile, 3.80[1.36–10.59]; 3^rd^ tertile, 7.30[2.50–21.29]; for MDA: 2^nd^ tertile, 3.08[1.13–8.42], 3^rd^ tertile, 6.39[2.24–18.25]). The 3^rd^ tertile of the 8-OHdG values was a high risk factor compared with the 1^st^ tertile (ORs [95% CI] for 8-OHdG: 2^nd^ tertile, 1.34[0.48–3.78], 3^rd^ tertile, 5.43[1.86–15.84]). These associations were not attenuated after the adjustment for age, smoking history, regular alcohol consumption, SBP, FBG, LDL, eGFR and pharmacological treatment of HTN, DM or dyslipidemia in model II [ORs (95% CI) for Isoprostane: 2^nd^ tertile, 4.22 (1.31–15.53); 3^rd^ tertile, 14.21 (3.34–60.56); CI] for MDA: 2^nd^ tertile, 4.22 (1.27–13.99), 3^rd^ tertile, 6.46 (1.91–21.83); for 8-OHdG: 2^nd^ tertile, 0.77 (0.22–2.64), 3^rd^ tertile, 4.45 (1.27–15.56)]. All p values for trend according to increment of ORs were below 0.01.

### The relationships between the oxidative damage markers and the risk of atherosclerosis (Table 3)

**Table 3:** Independent relationship among oxidative damage markers to carotid intima media thickness.

		Model A	Model B	Model C	Model D
		OR (95% CI)	OR (95% CI)	OR (95% CI)	OR (95% CI)
**Isoprostane,ng/mg creatinine**	1st tertile (<0.21)	1.00 -		1.00 -	1.00 -
	2nd tertile (<0.51)	4.72 (1.37–16.20)		3.37 (0.98–11.63)	3.93 (1.08–14.25)
	3rd tertile (≥0.51)	16.61 (3.44–80.15)		9.84 (2.12–45.70)	11.73 (2.30–59.74)
**MDA,μmol/g creatinine**	1st tertile (<0.10)		1.00 -	1.00 -	1.00 -
	2nd tertile (<0.20)		2.83 (0.80–10.06)	3.93 (1.15–13.45)	2.47 (0.66–9.20)
	3rd tertile (≥0.20)		4.74 (1.33–16.94)	3.59 (0.94–13.64)	2.38 (0.55–10.23)
**8-OHdG,μg/g creatinine**	1st tertile (<0.66)	1.00 -	1.00 -		1.00 -
	2nd tertile (<1.26)	0.58 (0.15–2.30)	0.81 (0.21–3.10)		0.77 (0.18–3.34)
	3rd tertile (≥1.26)	4.25 (1.05–17.16)	3.16 (0.83–12.10)		4.04 (0.94–17.50)

All models were adjusted for age, body mass index, smoking history, regular alcohol consumptions, systolic blood pressure, fasting plasma glucose, low density lipoprotein, glomerular filtration rate and pharmacological treatment of hypertension, diabetes and dyslipidemia

Abbreviations: Isoprostane, 8-iso-prostaglandin F2α; 8-OHdG, 8-hydroxy-2'-deoxyguanosine; MDA, malondialdehyde; OR (95% CI), odds ratio (95% confidence interval), OR (95% CI): odds ratio (95% confidence interval).

Multiple logistic regression models were constructed with covariates age, smoking history, regular alcohol consumption, SBP, FBG, LDL, eGFR and pharmacological treatment of HTN, DM or dyslipidemia. Two oxidative damage markers were incorporated into the same model. The ORs (95% CI) of the 3^rd^ tertile were 16.61 (3.44–80.15) for Isoprostane and 4.25 (1.05–17.16) for 8-OHdG in model A, 4.74 (1.33–16.94) for MDA and 3.16 (0.83–12.10) for 8-OHdG in model B, and 9.84 (2.12–45.70) for Isoprostane and 3.59 (0.94–13.64) for MDA in model C. When all the 3 oxidative damage markers were incorporated into the same multiple logistic regression model, MDA and 8-OHdG lost its significant association with CIMT. However, the associations with CIMT were still significant for Isoprostane (OR [95% CI]: 4.22 [1.31–13.53] in 2^nd^ tertile and 14.21 [3.34–60.56] in 3^rd^ tertile).

## Discussion

In this nested case-control study, increased levels of the oxidative damage markers were associated with the CIMT. Importantly, the associations between the oxidative damage markers and CIMT were still significant after controlling for the conventional risk factors, such as age, smoking history, regular alcohol consumption, SBP, FBG, LDL, eGFR and pharmacological treatment of HTN, DM or dyslipidemia. Furthermore, lipid peroxidation damage marker, Isoprostane, was independent from other oxidative damage markers when incorporated into one multiple logistic regression model to estimate the risk of atherosclerosis.

According to the oxidative modification hypothesis of atherosclerosis, the native state of LDL is not atherogenic [16]. However, entrapping of LDL in the sub-endothelial space leads to alteration of its surface net charge as a result of oxidative modification, which stimulates monocyte chemotaxis and inflammation [17]. Oxidized LDL is susceptible to uptake via the macrophage scavenger system, and this incorporation and accumulation in macrophages is a major cause of foam cell transformation and plaque formation [18]. Thus, free radical-initiated lipid peroxidation is a key mechanism of the development of atherosclerosis and inflammatory vascular damage. Based on these considerations, it can be expected that the association between oxidative damage markers and atherosclerosis is independent of the total blood level of LDL. Indeed, in the current study, the associations between the oxidative damage markers and the risk of atherosclerosis were still significant after controlling for conventional cardiovascular risk factors, such as age, smoking history, regular alcohol consumption, SBP, FBG, LDL, eGFR and pharmacological treatment of HTN, DM or dyslipidemia.

Isoprostane, the stable isomer of prostaglandin F2α, is formed non-enzymatically by a free radical attack on arachidonic acid, a lipid component of the cell membrane. Some studies compared the daily variation in the levels of Isoprostane in spot urine and 24-h urine samples, and observed no significant differences throughout the day in either samples [19]. In agreement with this, the mean levels of Isoprostane in morning urine samples were not significantly different from those in 24-h collection samples [20]. Therefore, the level of Isoprostane in the urine is widely used as the gold standard marker of lipid peroxidation [21]. We measured this level with HPLC-MS/MS, which has been reported to be one of the most reliable methods [22]. Several pathogenesis of cardiovascular are linked to Isoprostane excretion and formation. The pathogenic roles of Isoprostane on cardiovascular diseases were also proposed as vasoconstriction, platelet aggregation, angiogenesis and monocyte adhesion [23]. MDA is produced during the oxidative attack on lipoproteins and polyunsaturated fatty acids. Hence, MDA is one of the lipid peroxidation markers. However, these two lipid peroxidation markers (MDA and Isoprostane) were not independent of each other when estimating the risk of atherosclerosis in the same multiple logistic regression model (Table 3, model C). Furthermore, the Isoprostane more strongly related to CIMT compare to MDA did in model D, which incorporated all oxidative damage marker (Table 3).

Oxidative hydroxylation in the 8^th^ position of deoxyguanosine, which results in formation of 8-OHdG, is a common mutagenic DNA lesion [24]. Elevated levels of 8-OHdG are associated with various malignant conditions [25] and their prognoses [26]. Apoptosis, or programmed cell death, is induced by DNA damage, and endothelial cell death is an early event of atherogenesis that triggers plaque formation [27]. Apoptosis of vascular smooth muscle cells (VSMCs) is associated with the growth of plaques as a result of outward remodeling [28]. Moreover, it triggers intense intimal inflammation [29], which induces foam cell formation via the accumulation of lymphocytes. Formation of atherogenic lesions may be initiated in VSMC by mutational events, such as tumor cell growth after DNA damage [30]. Therefore, 8-OHdG is associated with atherosclerosis and plaque formation [24]. In the current study, the association between 8-OHdG and CIMT was significant after controlling for the lipid peroxidation markers (Isoprostane). That result suggested that this DNA damage marker has a somewhat different pathophysiological role. Xiang et al. [31] show increment of 8-OHdG related to number of coronary artery disease vessels. Hence, increment of 8-OHdG might predict the severity of cardiovascular diseases. Furthermore, that relationship was still significant after adjustment of age, sex, smoking hypertension, dyslipidemia, DM [31]. In the same context, our current study show the 8-OHdG were independently associated with the subclinical atherosclerosis in multivariable analysis including age, smoking history, regular alcohol consumption, SBP, FBG, LDL, eGFR and pharmacological treatment of HTN, DM or dyslipidemia.

Recently, Isoprostane also used to investigate the association between air pollutants exposure and oxidative modifications of lipoproteins. The lipid peroxidation detected by Isoprostane level was suggested as one of the key mechanism regarding to effect of air pollution on vascular diseases [32]. The complex combination of lipid peroxidation markers closely related to change of nano-plaque formation or size [33]. Hence, that clinical intervention study suggested that markers of lipid peroxidation might serve for monitoring of early diseases progress [33].

Several limitations were considered when interpreting the current results. The main limitation was the cross-sectional nature of our case-control study design, which prevents elucidation of cause-effect relationships. Furthermore, atherosclerotic lesions themselves produce oxidative stress, and this vicious circle contributes to the elevation of oxidative damage markers [17]. To minimize this effect, we excluded individuals who had a cardiovascular event, chronic hepatitis, osteoporosis, kidney disease, asthma, or any malignant disease, but we could not exclude patients with HTN, DM, and dyslipidemia. For example, glycemic disorders induce oxidative stress, and a chronic hyperglycemic status, such as in DM, is deeply associated with endothelial dysfunction, i.e., vascular disease [34]. Measuring of serum glucose level was only sampled after overnight fasting. Hence we have no data of post-prandial hyperglycemia or oral glucose tolerance test which status may induce oxidative stress. Furthermore we had no information on clinical severity and duration of DM, HTN and dyslipidemia, and family history of cardiovascular events which related to atherosclerosis events. Although the autoimmune diseases are related to oxidative stress and vascular damage, we cannot exclude that patient in current study. Therefore, to draw more firm conclusion about our aim of study, a more comprehensive and prospective cohort study is warranted to elucidate the causal relationship between oxidative stress and atherosclerosis. Furthermore, we have no information regarding the consumption of dietary vitamin supplements in our cohort, which could also reduce the levels of oxidative damage markers [35].

Finally, the relatively small sample size and the fact that the participants were from the same geographic area may limit the applicability of our results to the determination of the atherosclerosis risk in general population. Furthermore, because we used only male participant in current study, our results cannot be generalized to female population.

In summary, higher levels of the oxidative damage markers were correlated with CIMT. These associations remained significant after the adjustment for other conventional risk factors, such as LDL, BMI, smoking history, alcohol consumption, and MetS. Furthermore, the lipid peroxidation marker (Isoprostane) and the DNA damage marker (8-OHdG) were independent of each other when incorporated into the same multiple logistic regression model to estimate the risk of atherosclerosis.

## Supporting Information

S1 Data Text file containing all variables for our current study. (TXT)
